# Evaluation of equivalent dose from neutrons and activation products from a 15-MV X-ray LINAC

**DOI:** 10.1093/jrr/rrv045

**Published:** 2015-08-11

**Authors:** Isra Israngkul-Na-Ayuthaya, Sivalee Suriyapee, Phongpheath Pengvanich

**Affiliations:** 1 Department of Nuclear Engineering, Faculty of Engineering, Chulalongkorn University, Bangkok, Thailand; 2 Division of Therapeutic Radiology and Oncology, Faculty of Medicine, Chulalongkorn University, Thailand; 3 Division of Therapeutic Radiology and Oncology, Department of Radiology, King Chulalongkorn Memorial Hospital, Thailand

**Keywords:** equivalent neutron dose, activation products, Monte Carlo simulation, OSL

## Abstract

A high-energy photon beam that is more than 10 MV can produce neutron contamination. Neutrons are generated by the [γ,n] reactions with a high-Z target material. The equivalent neutron dose and gamma dose from activation products have been estimated in a LINAC equipped with a 15-MV photon beam. A Monte Carlo simulation code was employed for neutron and photon dosimetry due to mixed beam. The neutron dose was also experimentally measured using the Optically Stimulated Luminescence (OSL) under various conditions to compare with the simulation. The activation products were measured by gamma spectrometer system. The average neutron energy was calculated to be 0.25 MeV. The equivalent neutron dose at the isocenter obtained from OSL measurement and MC calculation was 5.39 and 3.44 mSv/Gy, respectively. A gamma dose rate of 4.14 µSv/h was observed as a result of activations by neutron inside the treatment machine. The gamma spectrum analysis showed ^28^Al, ^24^Na, ^54^Mn and ^60^Co. The results confirm that neutrons and gamma rays are generated, and gamma rays remain inside the treatment room after the termination of X-ray irradiation. The source of neutrons is the product of the [γ,n] reactions in the machine head, whereas gamma rays are produced from the [n,γ] reactions (i.e. neutron activation) with materials inside the treatment room. The most activated nuclide is ^28^Al, which has a half life of 2.245 min. In practice, it is recommended that staff should wait for a few minutes (several ^28^Al half-lives) before entering the treatment room after the treatment finishes to minimize the dose received.

## INTRODUCTION

A megavoltage photon beam can be generated by a linear accelerator and is widely employed in conjunction with radiation treatment technique to kill tumor cells in the cancer patient. High-energy photon beams (>10 MV) provide advantages over the lower-energy beams in terms of deeper penetration for greater depth dose, reducing skin dose, and decreasing peripheral dose due to smaller scatter. In clinical work, these advantages are quantified and applied to the calculation of dose distribution for patient treatment. However, the high-energy photons can also produce unwanted neutrons from the photoneutron [γ,n] interaction [1]. Neutrons are emitted from the interactions between photons and the nuclei of a high-atomic-number (high-Z) material when the photon energy is higher than the binding energy (5–15 MeV) of the nucleons [2]. The linear accelerator head components such as the target, primary collimators, flattening filter, secondary collimator, and multileaf collimators (MLCs) are made of high Z materials. Thus, undesirable neutrons can be produced from the accelerator head in the treatment room. During the treatment, the employed orientation and field size of the collimator jaws and the MLCs can also affect the number of neutrons produced. These neutrons can cause an increase in the dose received by the treated patient and the staff who access the treatment room, contributing to the risk of secondary malignancy in the patient in the future.

Various aspects of the photoneutron in the radiotherapy machine have long been studied [3–11]. Its characteristics have been determined using a variety of analytical techniques, and the results have shown large variations with respect to the equivalent neutron dose. There are two contributions to these variations. First, the studies used different instrumentations and methods of calculation for the photoneutron dose*.* Second, the equivalent neutron dose depends on the components of the linear accelerator head, the environment of the treatment room, and the energy of the photon beam.

Most studies have reported the neutron dose produced from the 18-MV photon beam, which is higher than the photon energy of our concern. Only a few reports have investigated the equivalent neutron dose produced by the 15-MV photon beam. Howell *et al.* measured the neutron spectrum using gold foil activation in Bonner spheres [3]. The average neutron energy was found to be 0.23 MeV for the 15-MV photon beam in the Varian LINAC machine. Zabihzadehet *et al.* studied the equivalent neutron dose and neutron spectra by Monte Carlo (MC) simulation, using a simple model of the LINAC head for estimation [4]. The equivalent neutron dose was found to be 4.1 mSv/Gy for the 15-MV photon beam. Due to the simplicity of the model used in the study, the accuracy of the MC calculation may be compromised.

For measurement, most studies used a thermoluminescent dosimeter (TLD) to determine the neutron dose equivalent. Nedaie *et al.* used TLD-600/TLD-700 dosimeters to measure the equivalent neutron dose and compared the results with the Monte Carlo simulation [5]. Not only the TLD but also other passive detectors such as gold foil, CR-39 track etch detectors and bubble detectors are commonly used for this purpose. Neutrons induce the nuclei in the gold foil to emit radiation which can be measured by a Ge(Li) detector system. The intensity of neutron flux can be estimated from the level of the induced radioactivity. CR-39 detector measurement is based on counting the number of tracks etched into the surface of the CR-39 detector after irradiation. Neutrons cannot generate any ionization in a detector directly. However, neutrons can produce charged particles such as protons and alpha particles that in turn cause ionization. For instance, recoil protons can be produced by the interactions of neutrons with the hydrogen atoms contained in a polyethylene radiator, and alpha particles can be produced from the ^10^B(n,α)^7^Li reactions in boron-loaded radiator. These charged particles can produce tracks on the detector. The dose is then evaluated by counting the number of tracks. Bubble detectors have tiny droplets of superheated liquid dispersed throughout a clear polymer. After the neutrons strike a droplet, the droplet immediately vaporizes, forming a visible gas bubble trapped in the gel. The number of bubbles yields a direct measurement of the tissue-equivalent neutron dose. Fujibuchi *et al.* measured the secondary neutron dose for the 10-MV and 15-MV X-ray LINAC using the gold foil and CR-39 detectors [12]. The results showed that the neutron dose for the 15-MV LINAC was 10 times higher than the dose for the 10-MV LINAC because the cross-section of the photonuclear reaction increased in the higher-energy LINAC. The Viamonte *et al.* study showed that the OSL system was suitable for dosimetry-related measurement in high-energy photon beams [13]. Their results showed good agreement with the ionization chamber and the diode measurements at similar positions. Yukihara *et al.* investigated the OSL detectors that were produced by Landauer [14]. The reproducibility of the OSL signal for multiple irradiations was found to be in the order of 1%. OSL dosimeters were therefore suitable for dosimetry measurement in radiotherapy. However, there has been no report of using Optically Stimulated Luminescence (OSL) to measure the neutron dose equivalent in the medical LINAC for the 15-MV X-ray beam.

Inside the treatment room, not only neutrons are generated. Gamma rays are also generated as the result of interactions between the photoneutron and the high-Z materials in the LINAC head and the treatment room through the [n,γ]-type reaction. Since the gamma rays can be emitted around the treatment room, this undesirably increases the dose to the patient and the staff who access the treatment room during and shortly after the treatment. Rawlinson *et al.* [15] and Fischer *et al.* [16] reported many activation products that varied in type, quantity and location from different LINAC machines and room geometries. The annual dose that a radiation therapist received during routine work was in the range of 0.7–5 mSv per year [17–20].

The gamma spectrum of the induced activity should be further investigated in order to identify the types and quantities of the isotopes involved. The excess dose should be quantified in order to correctly implement radiation protection policy in the treatment room, i.e. the occupancy dose should not exceed the dose limit prescribed in *ICRP Publication 103* [21].

The objective of this work is to investigate the neutron spectra and dose equivalent around the LINAC head at various distances, as well as the effect of using different field sizes for the treatment. The MC simulation has been employed to estimate the photoneutron spectra in the LINAC machine. The simulation has been set up to match the machine's geometry and position in an actual treatment room at King Chulalongkorn Memorial Hospital. The spectrum of photoneutrons has been investigated at the target, collimator and isocenter of the machine. The neutron dose obtained from the simulation has also been compared with the actual measurement using OSL. The activation product and gamma dose were investigated inside the treatment room by measurement.

## MATERIALS AND METHODS

### Monte Carlo calculation

**Fig. 1.**
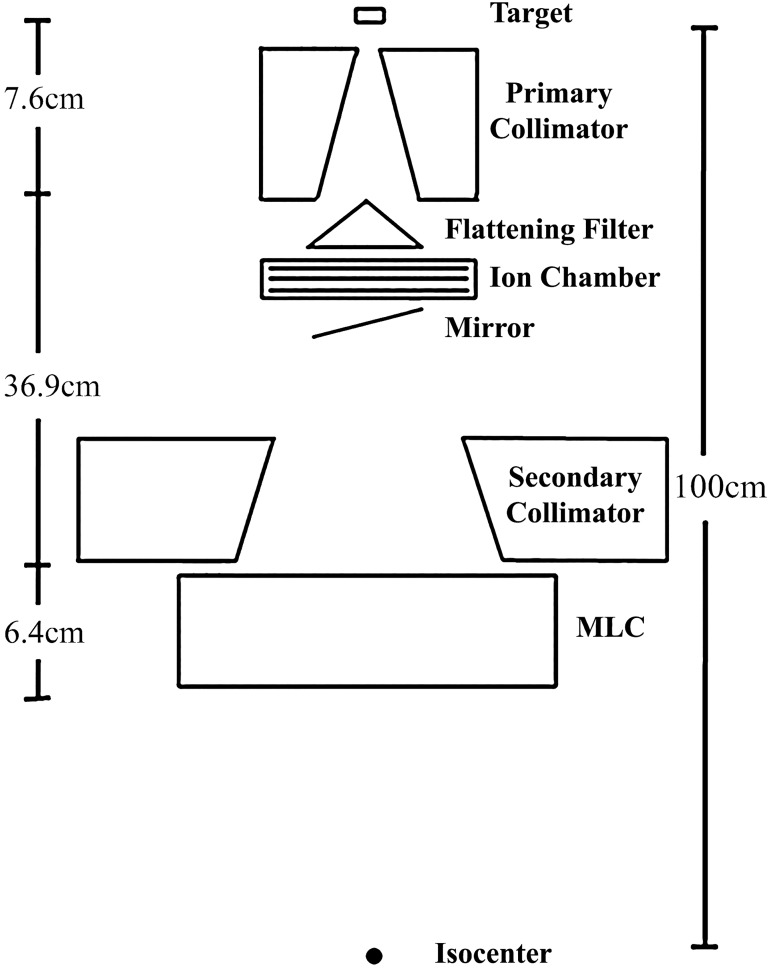
Model of the linear accelerator head used for MC simulation.

The Monte Carlo calculation in this study is based on the MCNP code version 5 (MCNP5) and has been employed to calculate the neutron spectrum and the equivalent neutron dose. Several components of the LINAC head, including the target, primary collimator, flattening filter, secondary collimator and MLCs, were simulated based on the 15-MV photon beam source Clinac 23EX machine (Varian Medical Systems, Palo Alto, CA, USA), as shown in Fig. 1. Their geometrical data, which was obtained from the manufacturer, was input into the MCNP5 code.

**Fig. 2.**
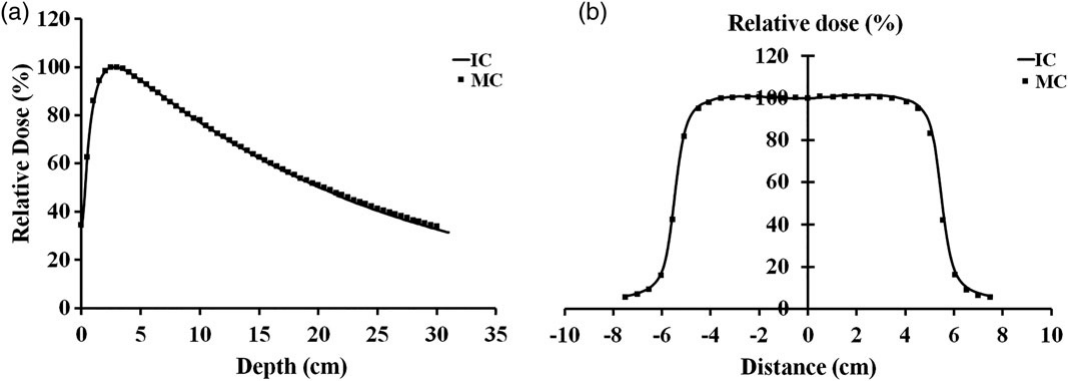
Percentage depth–dose curve and beam profile of 15-MV X-rays compared between Monte Carlo simulation and ionization chamber. (a) PDD opening 10 × 10 cm^2^ field size. (b) Beam profile opening 10 × 10 cm^2^ at 10 cm depth.

A primary source of electrons was used in Gaussian energy distribution mode for simulation of the 15-MV photon beam. The energy cutoff was <0.1 MeV to reduce calculation time for photons because the photon energy must be >10 MeV to produce photoneutrons. The incident electron energies were adjusted in the MCNP code until the percentage depth dose (PDD) and the beam profile matched with the beam data measured by the ionization chamber (0.13 cm^3^ volume) within 1%. The final incident electron energy used in the simulation was 13.5 MeV. Comparisons between the simulated values and the ionization chamber measurements of the PDD and the beam profiles are shown in Fig. 2. Neutron spectrum and flux were calculated using 10^7^ simulate particles. The photon–electron–neutron transport mode was used for running the simulation. The simulated detection volume had a cylindrical shape with 1-cm radius and 1-cm thickness. The human body was modeled with a water equivalent phantom sized 30 × 30 × 30 cm^3^. The neutron flux was also computed in F4 tally mode, and was converted to equivalent neutron dose using the flux-to-dose conversion factor from *ICRP Publication 74* [22]. The calculation uncertainty in each energy bin was <3%. The MCNP5 code was set to determine the neutron spectrum and the average neutron energy. The neutron spectrum was simulated at the target, collimator and isocenter of the beam. The neutron dose at each position was compared with the OSL measurement.

### Optically Stimulated Luminescence measurement

The Optically Stimulated Luminescence (OSL) used for photon measurement in this study consisted of the single crystal carbon doped aluminum oxide (Al_2_O_3_:C). The OSL technology is the newest advancement in passive radiation dosimetry. The method employs the electrons trapped between the valence and the conduction bands in the crystalline structure. The trapping sites are imperfections of the lattice impurities. The ionizing radiation produces electron–hole pairs in which electrons are in the conduction band and holes are in the valence band. The electrons, which have been excited to the conduction band, may become entrapped in the electron or hole traps. Under the stimulation of light, the electrons may free themselves from the trap and get into the conduction band. From the conduction band, they may recombine in hole traps. If the center with the hole is a luminescence center, emission of light will occur. The read-out process uses a light emitting diode (LED) array to stimulate the detector. The light emitted from the OSL detector is then measured by a photomultiplier tube (PMT) counting system (Inlight system, Landauer). The signal from the tube is then used to calculate the dose. The N-type of OSL (OSLN) dosimeter is another type of detector that can measure both neutron and photon beams. It is capable of measuring neutron energies between 40 and 5000 keV. The OSLN contains Al_2_O_3_:C coated with ^6^Li_2_CO_3_. Neutrons interacts with the ^6^Li and produce both tritium and alpha particles following Equation 1. These particles give up their energy to the Al_2_O_3_:C in a process that generates a stored charge. OSLN detectors can be annealed to remove existing measurement using the Landauer's model 50A automatic annealer. So, OSLN can be repeatedly used for measuring neutron dose.

(1) $${}_{3}^{6}Li+{}_{0}^{1}n\to {}_{1}^{3}H+\;\;{}_{2}^{4}\propto \frac{Q-\;value}{4.78\;MeV}\;$$

**Fig. 3.**
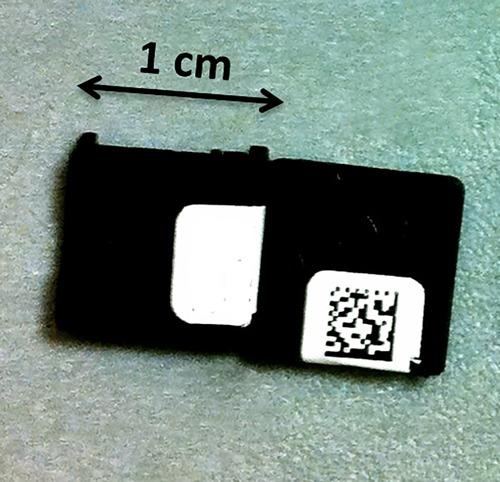
The nanoDot type of OSL using for measurement.

The OSL detectors were placed on the solid water phantom at various positions for measurement during delivery of the 15-MV photon beam. For mixed-beam irradiation, OSL dosimeters were used to measure the photons only, while OSLN dosimeters were used to measure both photons and neutrons. The neutron dose was determined by subtracting the photon signal measured by the OSL dosimeters from the signal measured by the OSLN dosimeters. Both types of OSL should be used at the same time for measurement. The nanoDot type of OSL, which was employed for this study, is shown in Fig. 3. The maximum relative sensitivity of OSL varied about 3.1% of one standard deviation [23]. At each position, the measurement was repeated five times, and the average reading in units of mSv per photon Gray (mSv/Gy) was reported. The OSL detectors were calibrated with a ^137^Cs gamma source, and the OSLN detectors were calibrated with a ^252^Cf neutron source because the energy range of ^252^Cf covered the energy range of the photoneutrons generated inside the treatment head of the LINAC.

### Neutron dose equivalent comparison

The equivalent neutron doses in the units of mSv/Gy obtained from simulation and measurement were compared under various conditions. In this study, the combinations of field size (collimator jaw), SSD, off-axis distance, and field size (MLCs) shown in Table 1 have been investigated.

**Table 1.** The various parameters for simulation and measurement

Experiment	Field size (cm^2^)	SSD (cm)	Off-axis distance (cm)	Field size by MLC (cm^2^)
1	2 × 2	100	0	N/A
	5 × 5			
	10 × 10			
	15 × 15			
	30 × 30			
2	10 × 10	80	0	N/A
		100		
		150		
		200		
3	10 × 10	100	0	N/A
			100	
			150	
			200	
4	30 × 30	100	0	2 × 2
				5 × 5
				10 × 10
				15 × 15

### Activation products and dose rate investigation

Neutrons can induce the activation products, which emit gamma-rays through the [n,γ] reaction with materials inside the treatment machine. The activation products were investigated by a gamma spectrometer system. A radionuclide identifier system was used to determine the emitting isotopes. The portable radionuclide identifier utilized the 3″ × 3″ sodium iodide (NaI) scintillation detector (SAM 940 eagle, Berkeley Nucleonics Corporation, San Rafael, CA, USA). The gamma spectrum was collected at 100 cm SSD after finishing the irradiation at 1000 MU, while the gamma dose rate in units of mSv/h was measured using a survey meter. The survey meter is Victoreen model 190 with an **energy-compensated Geiger-Mueller (GM) Probe Model 90-12** (Cardinal Health, Dublin, OH, USA). The detector was put on top of the treatment couch. The gantry was set at an angle of 0 degrees. The spectrum was measured inside the light field when the collimator jaws were opened to 10 × 10 cm^2^. The 15-MV photon beam was delivered at the dose rate of 400 MU/min, i.e. a clinical dose rate. Gamma spectra were analyzed in order to identify the isotopes and the percentage of yield of each isotope. Then, gamma spectrum and dose rate were recorded right after the irradiation. The recording time was 5 min. The measurement of the gamma dose rate was repeated at the same position at 5 min, 1 h, 12 h and 24 h after the treatment in order to investigate and analyze the long half-life isotopes. The gamma dose rate was investigated for the purpose of the radiation protection and safety of patient and staff.

## RESULTS

### Neutron spectra

**Fig. 4.**
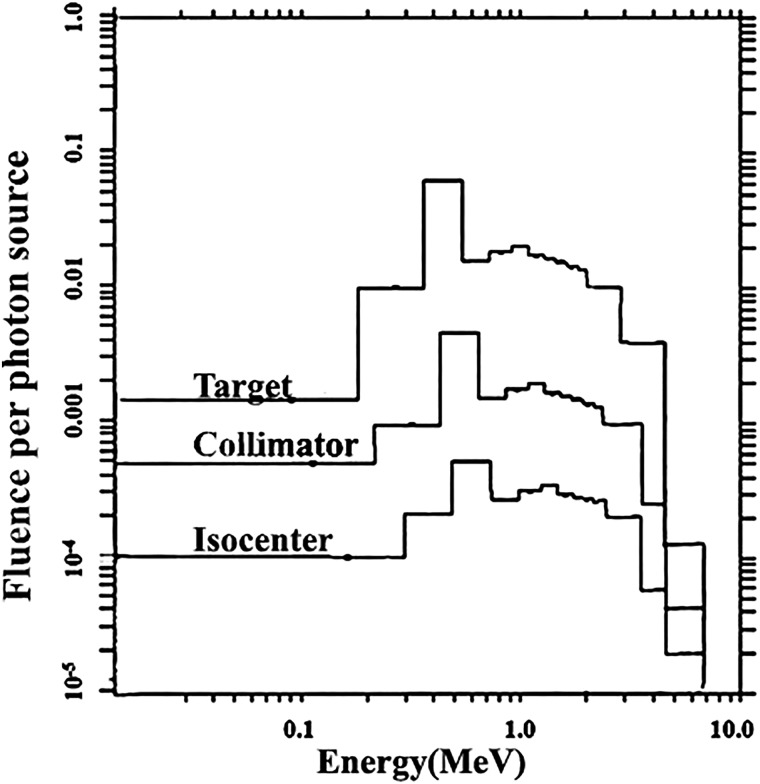
Neutron spectra simulated by Monte Carlo Method at the target, the primary collimator and the isocenter positions.

The neutron spectrum was simulated for the 15-MV photon beam modeled after the Varian 23EX linear accelerator. The number of initial electrons used for the simulation was 10 million. The components of the Linear accelerator head were created according to the manufacturer information, but it didn't include the room geometry for decreasing time calculation. Neutron spectrums were calculated by MCNP5 code at the target, collimator jaws, and isocenter of the field. The neutron spectrum was shown in Fig. 4. The average neutron energy was ∼0.25 MeV, and the maximum peak was located at ∼0.5 MeV.

### Equivalent neutron dose

The neutron dose equivalent measured by OSLN was compared with the value calculated by MCNP at various positions inside treatment room.

For different field sizes, the equivalent neutron doses calculated and measured are shown in Table 2. OSL dosimeters were irradiated at 100 cm SSD on top of the 30 × 30 × 30 cm^3^ solid water phantom. The ranges of measured and calculated neutron dose for 2 × 2, 5 × 5, 10 × 10, 15 × 15 and 30 × 30 cm^2^ were 3.79–6.75 mSv/Gy and 4.75–6.31 mSv/Gy, respectively.

**Table 2.** Equivalent neutron doses for different field sizes

Field size (cm^2^)	Measurement (mSv/Gy)	Calculation (mSv/Gy)
2 × 2	3.79	3.45
5 × 5	4.09	3.79
10 × 10	5.39	4.34
15 × 15	5.61	4.53
30 × 30	6.75	6.31

The equivalent neutron dose was also investigated for different SSDs with the same field size of a 10 × 10-cm^2^ beam. The measurement and calculation at different SSD positions yielded the range of neutron dose between 2.41 and 7.45 mSv/Gy and between 1.95 and 6.98 mSv/Gy, respectively, as shown in Table 3.

**Table 3.** Equivalent neutron doses at different distances

SSDs (cm)	Measurement (mSv/Gy)	Calculation (mSv/Gy)
80	7.45	6.98
100	5.34	4.53
150	4.29	3.97
200	2.41	1.95

The equivalent neutron doses at the in-field and out-of-field distances are shown in Table 4. The neutron doses measured and calculated at the isocenter were 5.39 and 4.34 mSv/Gy, respectively. At 100 cm out-of-field, the neutron doses measured and calculated were 0.42 and 0.75 mSv/Gy, respectively.

**Table 4.** Equivalent neutron doses at in-field and out-of-field distances

Distance (cm)	Measurement (mSv/Gy)	Calculation (mSv/Gy)
Isocenter (0)	5.39	4.34
100	0.42	0.75
150	0.21	0.62
200	0.15	0.43

The comparison between the measured and calculated equivalent neutron dose was performed at various field sizes opened by the MLC while the collimator jaws were fixed at 30 × 30-cm^2^ field size. The results are shown in Table 5. The equivalent neutron dose was increased by closing the MLC position.

**Table 5.** Equivalent neutron dose for different field sizes opened by MLCs

Field size (cm^2^)	Measurement (mSv/Gy)	Calculation (mSv/Gy)
2 × 2	9.75	9.25
5 × 5	7.02	6.51
10 × 10	4.99	4.19
15 × 15	4.32	3.84

### Activation products

**Fig. 5.**
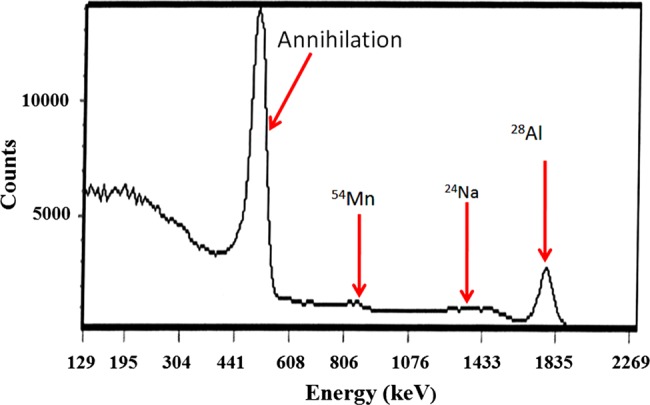
Gamma spectrum generated by activation products inside the linear accelerator room.

Activation products are generated when the photoneutrons interact with the components of the LINAC head. These activation products can emit gamma rays that may lead to exposure of the occupants. The gamma spectrum measured by a spectrometer showed the gamma peaks corresponding to several activation products in Fig. 5. Spectrum analysis revealed dominant radioisotopes, i.e. ^28^Al, ^24^Na, ^54^Mn and ^60^Co, of which the highest peak of the spectrum corresponded to ^28^Al. The characteristics of each isotope are displayed in Table 6. The spectrum showed the annihilation peak at 511 keV as a result of beta plus decay from ^54^Mn.

**Table 6.** The characteristic of activation products generated inside the linear accelerator room

Nuclides	Gamma energy (keV)	Half-life	Dose (mSv/h)	% Activity
^28^Al	1779	2.24 min	3.565	86.10
^24^Na	1369	15 h	0.548	13.24
^54^Mn	847, 1811	2.58 day	0.018	0.43
^60^Co	1253	5.27 year	0.009	0.20

The equivalent gamma dose rate of 4.14 µSv/h was measured at 100 cm SSD immediately after irradiation at 1000 MU. The doses were emitted by the activation products inside the treatment room. The equivalent dose rate decreased to 0.65 µSv/h after 5 min waiting time. The equivalent dose rate was <0.001 µSv/h (background dose) after a decay time of >1 h.

## DISCUSSION

From the neutron spectrum in Fig. 4, the average neutron energy was ∼0.25 MeV, and the maximum peak was located at ∼0.5 MeV. The average energy of a neutron reported by Howell *et al.* [3] and Zabihzadeh *et al.* [4] was 0.23 MeV and 0.20 MeV, respectively. The neutron spectrum was quite the same shape at each component of LINAC head such as the target, primary collimator, and isocenter, but the intensity differed in each position. The neutron flux showed the most intensity at the target. The intensity of the neutron flux decreased when the position was far from the target.

For fast neutrons (>10 keV), the predominant moderating effect came from inelastic scattering in high atomic number materials located inside the components of the LINAC head. But this interaction did not occur at energy below the lowest excited state of the material. While it was difficult to estimate the neutron energy loss due to inelastic scattering, the loss was minimal since the energy of neutrons produced in the treatment room was mostly less than the lowest excited state. This was the reason why the energy of the neutron did not reduce when it passed from the target to the isocenter.

The beam-hardening effect of the photon occurred inside the LINAC head, causing a slight increase in the average neutron energy at the isocenter. A higher-energy photon can produce higher neutron energy. The neutron flux intensity reduced from the target to the isocenter because the number of photoneutrons generated inside the LINAC head at the target was larger than those at the other components, and the neutron attenuation varied with distance.

For different field sizes, the equivalent neutron dose increased for larger field size, as shown in Table 2. The results agreed with the equivalent neutron dose reported in other studies. Zabihzadeh *et al.* [4] reported the equivalent neutron dose of 4.1 mSv/Gy at the isocenter for the 40 × 40-cm^2^ field size calculated by Monte Carlo simulation. Chibani *et al.* [2] reported the equivalent neutron dose of 13.3 mSv/Gy for the 10 × 10-cm^2^ field size calculated by the MCNPX code in Varian machine. The equivalent neutron dose was also investigated for different SSDs. The measurement and calculation are shown in Table 3. The neutron dose decreased as the SSD increased.

For Table 4, the neutron dose at out-of-field was less than at the isocenter by several times because the maximum intensity of the neutron fluence was generated at the target of the linear accelerator head. Therefore, the neutron fluence was absorbed by the shielding components of the machine. Zabihzadeh *et al.* [5] reported the equivalent neutron dose of 4.1 mSv/Gy at the isocenter and 0.79 mSv/Gy at a distance of 100 cm out-of-field from the isocenter for the 40 × 40-cm^2^ field size in the 15-MV photon beam. The value agreed well with our study. The comparison between the measured and calculated equivalent neutron dose was performed at various field sizes opened by the MLC while the collimator jaws were fixed. The equivalent neutron dose increased by closing the MLC position. When the MLCs were closed, the dominance of neutrons can be generated more in the MLCs, which are made from tungsten material. Meo *et al.* [24] reported the neutron yield of each component, which agreed well with our study.

For in-field positions, the equivalent neutron doses calculated by the MCNP5 were lower than the corresponding values measured by the OSLN. Neutrons can scatter from the floor, wall and ceiling of the treatment room, as well as from the equipment around the treatment room. In order to limit the calculation time, the MC simulation did not include every environmental geometry inside the treatment room. Thus, the calculated neutron dose was consistently less than the measured neutron dose at every in-field position.

For out-of-field, the neutron doses calculated by the MCNP5 were higher than the measured doses because the calculation underestimated the contribution of the shielding components. The shielding thickness of the linear accelerator head was not included in the simulation due to lack of information from the manufacturer.

In this study, the neutron spectrum from the 15-MV photo beam was investigated by the MCNP5 code. The dominant neutron flux is generated at the beam target. The equivalent neutron dose was between 3.45 and 9.75 mSv per photon Gy at the isocenter of the field. The equivalent neutron dose was found to be highest when the collimator jaws were fixed at 30 × 30 cm^2^ and the MLC was at 2 × 2 cm^2^. At 100 cm out of field, the neutron dose was 10 times less than the dose in field. The calculated and measured neutron doses were in agreement in every position compared. The MCNP5 code can be used to determine the equivalent neutron dose inside the treatment room. This finding helps to predict the neutron dose, and provides important information for determining radiation protection policy for patients and staff working at the treatment room.

Activation products are generated when the photoneutrons interact with the components of the LINAC head. These activation products can emit gamma rays that may lead to exposure of the occupants. Determination of the percentage yield of each radioisotope found that the dominant activation product was ^28^Al immediately after the irradiation. Several radioisotope products disappeared in a few minutes after the irradiation because of their short half-lives. One hour after the irradiation, ^54^Mn was the dominant product. ^24^Na was the dominant product between 12 and 24 h following the irradiation. After 24 h, the dose rate was nearly equivalent to that of the background from the weak radioisotopes of ^60^Co and other particles' long half-life radioisotopes. Other long half-life radioisotopes have been reported by Fischer *et al.* [16]. All of these major radioisotopes were generated by neutron capture interactions because photoneutrons interacted with materials inside the treatment head and room.

As already mentioned above, the dominant activation product immediately after the irradiation was ^28^Al. Since ^28^Al is a short half-life isotope, protection against it can be easily implemented by allowing it to decay for a short period of time before occupying the room after the irradiation. For other isotopes (^54^Mn, ^24^Na and ^60^Co) that have longer half-lives, it is found that the personal gamma dose received is ∼0.78 mSv/year which is well under the recommended occupational dose limit [21]. The annual dose limit for occupants is 20 mSv/year. Thus, it is recommended that the therapist or staff should wait for a few minutes before entering the treatment room after irradiation, and stay for a short period of time. That way, the radiation exposure can be minimized.
